# Retrieval of chromophore concentration changes in a digital human head model using analytical mean partial pathlengths of photons

**DOI:** 10.1117/1.JBO.29.2.025004

**Published:** 2024-02-28

**Authors:** Demián A. Vera, Héctor A. García, Nicolás A. Carbone, María Victoria Waks-Serra, Daniela I. Iriarte, Juan A. Pomarico

**Affiliations:** CIFICEN (UNCPBA - CICPBA - CONICET), Tandil, Argentina

**Keywords:** functional near infrared spectroscopy, multilayered media, continuous wave, Monte Carlo, head model, chromophores

## Abstract

**Significance:**

Continuous-wave functional near-infrared spectroscopy has proved to be a valuable tool for assessing hemodynamic activity in the human brain in a non-invasively and inexpensive way. However, most of the current processing/analysis methods assume the head is a homogeneous medium, and hence do not appropriately correct for the signal coming from the scalp. This effect can be reduced by considering light propagation in a layered model of the human head, being the Monte Carlo (MC) simulations the gold standard to this end. However, this implies large computation times and demanding hardware capabilities.

**Aim:**

In this work, we study the feasibility of replacing the homogeneous model and the MC simulations by means of analytical multilayered models, combining in this way, the speed and simplicity of implementation of the former with the robustness and accuracy of the latter.

**Approach:**

Oxy- and deoxyhemoglobin (HbO and HbR, respectively) concentration changes were proposed in two different layers of a magnetic resonance imaging (MRI)-based meshed model of the human head, and then these changes were retrieved by means of (i) a typical homogeneous reconstruction and (ii) a theoretical layered reconstruction.

**Results:**

Results suggest that the use of analytical models of light propagation in layered models outperforms the results obtained using traditional homogeneous reconstruction algorithms, providing much more accurate results for both, the extra- and the cerebral tissues. We also compare the analytical layered reconstruction with MC-based reconstructions, achieving similar degrees of accuracy, especially in the gray matter layer, but much faster (between 4 and 5 orders of magnitude).

**Conclusions:**

We have successfully developed, implemented, and validated a method for retrieving chromophore concentration changes in the human brain, combining the simplicity and speed of the traditional homogeneous reconstruction algorithms with robustness and accuracy much more similar to those provided by MC simulations.

## 1 Introduction

Continuous-wave functional near-infrared spectroscopy (CW-fNIRS) has gained attention in the last three decades due to its capability to assess changes in chromophore concentrations non-invasively in the human brain that are, in turn, related to neuronal activity in the cortex.^1^^–^^5^ Usually, this is carried out with the use of relatively inexpensive and portable devices that utilize CW sources (lasers or LEDs) to shine light into the subject’s head, and one to several detectors, at a distance ρ from the source, that collect the diffusely reflected light carrying information of metabolic changes that occur in the cortex as well as other regions of the head.^5^^,^^6^ Due to all this, applications of CW-fNIRS have reached several subfields in medicine, neurology, and neuroscience.^7^^–^^12^

The main objective of CW-fNIRS is to retrieve concentration changes of particularly two chromophores: oxygenated and deoxygenated hemoglobin (usually abbreviated as HbO and HbR, respectively); to do so, a minimum of two different wavelengths must be considered, at both sides of the isosbestic point (∼800  nm) of HbO and HbR.^13^ However, some difficulties may arise that mask the desired information that comes from the cortex. On one hand, NIR light must travel through various tissues before reaching the cortex; in fact, most of the energy is absorbed in the scalp (∼76%) and the skull (∼20%);^14^ on the other hand, there also are changes in both oxy- and deoxygenated blood that take place in the scalp, the region that is in contact with the optodes (that is, sources or detectors).^15^^,^^16^ To separate the data coming from the scalp and that originated in the cortex, several strategies have been thought. The most extended, and considered now as a mandatory experimental procedure, is the use of the so called short separation (SS) channels (detectors placed at shorter distances from the source), that permits the subtraction of information that comes mostly from the scalp, leaving the cortical signal almost free from the influence of the superficial layers.^17^^,^^18^ Currently, this method is widely used with different degrees of success,^19^^–^^21^ and although the retrieved signal is considered to be a mixture of subsignals from scalp and gray matter, the head model is still taken as homogeneous; that is, the theoretical description considers that the metabolic changes take place in all the volume at the same time. This simplification is rather far from reality, where one can expect that changes in chromophore concentrations come from different tissues.

In 1993, Hiraoka et al.^22^ proposed a two-layered model to distinguish the signals arising from a superficial extracerebral layer and a deeper cortical layer. In that work, they managed to link changes in optical density with relative absorption changes and the corresponding mean partial pathlengths (MPPLs) in each of both layers, which were obtained by means of Monte Carlo (MC) simulations. Later, other contributions^23^^–^^25^ extended Hiraoka’s method to the time domain (TD), so as to consider the first and second statistical moments of the distributions of time of flight of photons, showing that the retrieval’s accuracy in the deepest layers increases with the order of the moment. Recently, Ortega-Martínez et al.^26^ showed a performance comparison between reconstructions using traditional CW-fNIRS and moments-based TD-fNIRS, arriving at the conclusion that the latter is more accurate since TD data carry more information on photon migration than its CW counterpart. At this point, it is worth mentioning that all the cited works, as well as many others,^27^^–^^31^ made use of multilayered models of the human head, but basing their results on MC simulations.

Another extended fNIRS imaging technique is diffuse optical tomography, which intends to recover a map of the activated regions of the brain that are under study. To this end, it is usual to cover the head with a high-density probe. To simulate data coming from the cortex, a very useful tool is the propagation of light by means of the so-called finite element method (FEM).^32^

In a previous work, some of the authors^33^ obtained analytically computed MPPLs for multilayered media of up to four layers. The main advantage of this approach is that their computation takes much less time than with MC simulations and FEM methods, even without necessarily having demanding hardware capabilities such as dedicated graphic cards (GPUs). This study was then extended in 2023^34^ for media with an arbitrary number of layers. In another work from 2023,^21^ we also studied the analytical reconstruction of absorption changes in semi-infinite layered media with planar interfaces generating data by means of MC simulations as well as in experiments with phantoms. Here it was shown that the two-layered and the four-layered retrievals outperform the homogeneous reconstruction, even with the latter considering short channels.

In the present work we extend the validation described in the previous paragraph^21^ by generating HbO and HbR concentration changes in a five-layered MRI meshed model of the human head via MC simulations,^35^ and retrieving the proposed concentration changes using both, a two- and a five-layered model of the head. Our reconstructions are then compared to those obtained using the standard homogeneous approach, which considers short channels to take into account the undesired contribution of the superficial layers; in the case of the five-layered reconstruction, we also show the ideal retrieval performed using the MC-based MPPLs. Results suggest that using layered models is much more appropriate to retrieve concentration changes than using homogeneous models, even when the latter implement the use of short channels. In the particular case of using analytical MPPLs, the computation times are much more similar to those involved in the homogeneous models calculations, while the accuracy of the retrieved values is comparable to those obtained with MC reconstructions.

This contribution is organized as follows: Section 2 introduces the theoretical background needed to obtain the concentration changes using a layered model. Section 3 first describes the main aspects of the MC simulation process, together with the geometry employed; then, details on two distinctive and plausible situations are introduced as proposals for the concentration changes, as well as the reconstruction obtained with the homogeneous and the analytical layered approaches. The main results are presented in Sec. 4, and finally, the conclusions are summarized in Sec. 5.

## 2 Theoretical Concepts

### 2.1 Light Propagation in Turbid Media

Light propagation in media presenting absorption and scattering can be fully described by the radiative transfer equation (RTE)^36^

s^·∇I(r→,s^)+(μa+μs)I(r→,s^)=μs∫S2f(s^·s^′)I(r→,s^′)ds^′+ε(r→,s^), (1)

where I(r→,s^) is the radiance (${\mathrm{Wm}}^{-2}$), s^ represents the direction of propagation of photons, and $\mu_{a}$ and $\mu_{s}$ the absorption and scattering coefficients, respectively. f(s^·s^′) is the phase function, that accounts for the probability of photons that comes from direction s^ to be scattered in the direction s^′. Finally, ε(r→,s^) is the source term. Although this is an exact equation for describing the photon behavior, it is only solvable in an analytical manner in media with certain degree of symmetry. However, if the studied medium is turbid, i.e., the probability of scattering events are at least two orders of magnitude greater than the absorption events, the RTE can be approximated, under the so-called diffusion approximation (DA), by a diffusion equation (DE)^36^

$$(-D\nabla^{2}+\mu_{a})\Phi (\vec{r})=S(\vec{r}),$$ (2)

where $D=1/3\mu_{s}^{\prime }$ is the diffusion coefficient, $S(\vec{r})$ is the source term, and $\Phi (\vec{r})$ represents the fluence (with units of ${\mathrm{Wm}}^{-2}$), which is defined as the average of the radiance over the solid angle Ω

Φ(r→)=∫ΩI(r→,s^)dΩ. (3)

### 2.2 Modified Beer–Lambert Law

**Fig. 1:**
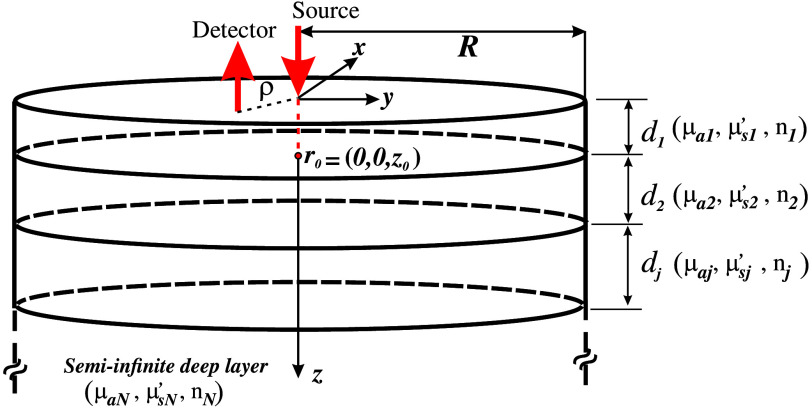
Scheme of a semi-infinite N-layered turbid cylinder under a reflectance measurement configuration. A stack of N−1 discs j=1,2,…,N−1 of radius R and thicknesses $d_{j}$, on top of an N’th compartment of infinite thickness, is considered. The light from the source impinges at r=(0,0,0). Due to the diffusive regime, the scattering becomes completely isotropic at $r_{0}=(0,0,z_{0})$, with $z_{0}=1/\mu_{s,1}^{\prime }$. Finally, the diffusely reflected light emerging from the medium is collected at a distance ρ from the illumination point.

The modified Beer–Lambert law is used to derive changes in tissue optical properties based on CW diffuse optical intensity measurements. It relates differential light transmission changes (in any geometry) to differential changes in tissue absorption. According to it, light attenuation A(λ,ρ) inside an N-layered turbid medium in a reflectance configuration (Fig. 1) can be modeled as follows:^22^^,^^23^^,^^37^

$$A(\lambda ,\rho )=-\log[\frac{I(\lambda ,\rho )}{I_{0}(\lambda ,\rho )}]=\overset{N}{\underset{j=1}{\sum }}L_{j}(\lambda ,\rho )\Delta \mu_{a,j}(\lambda ),$$ (4)

where $\Delta \mu_{a,j}$ is the absorption coefficient’s change taking place in layer j; $I_{0}(\lambda ,\rho )$ and I(λ,ρ) are, respectively, the baseline signal (before any absorption change takes place) and the detected signal during the whole experiment session (both measured at a wavelength λ and at a distance ρ from the source); and $L_{j}(\lambda ,\rho )$ is the photon MPPL in layer j. Technically speaking, $L_{j}(\lambda ,\rho )$ also depends on the layer thicknesses, $d_{j}$, the corresponding refractive indexes, $n_{j}$, and on the reduced scattering coefficient of each layer, $\mu_{s,j}^{\prime }=\mu_{s,j}(1-g_{j})$, $g_{j}$ being the anisotropy factor of layer j. This quantity represents the mean value of the cosine of the azimuthal scattering angle and, for brain tissue, it is usually taken as 0.9^38^ (please note that when working under the DA regime any information about g is implicitly considered within the reduced scattering coefficient). Nevertheless, in fNIRS applications it is usually assumed that all of these parameters remain constant, implying that any measurable change is solely attributed to changes in the absorption coefficients $\mu_{a,j}$. This means that, in principle, $L_{j}$ implicitly depends on the $\mu_{a,j}$ of the different layers (through the wavelength λ), and these absorption coefficients are none but the baseline values with respect to which the absorption changes take place.^25^ Please note that by setting N=1 in Eq. (4) the attenuation for a homogeneous medium is achieved.

Changes in chromophores concentrations can be linked to A(λ,ρ) by the following relation:

$$\Delta \mu_{a,j}(\lambda )=\varepsilon_{\mathrm{HbO}}(\lambda )\Delta [\mathrm{HbO}{]}_{j}+\varepsilon_{\mathrm{HbR}}(\lambda )\Delta [\mathrm{HbR}{]}_{j},$$ (5)

where $\varepsilon_{\mathrm{HbO}}(\varepsilon_{\mathrm{HbR}})$ is the molar extinction coefficient of HbO (HbR) that depends on the wavelength λ, and Δ[HbO] (Δ[HbR]) represents the concentration changes in oxygenated (deoxygenated) blood. Now, replacing expression Eq. (5) in Eq. (4), we get

$$A(\lambda ,\rho )=\underset{j}{\sum }[\varepsilon_{\mathrm{HbO}}(\lambda )\Delta [\mathrm{HbO}{]}_{j}+\varepsilon_{\mathrm{HbR}}(\lambda )\Delta [\mathrm{HbR}{]}_{j}]\cdot L_{j}(\lambda ,\rho ).$$ (6)

So far, this problem has infinite solutions, considering that there are two unknowns, HbO and HbR, for each layer j, and just one equation. However, two modifications should be done in order to find a system with an unique solution: (a) adding a different wavelength will lead to another analogous equation that can be employed to solve the system

$$A(\lambda_{1},\rho )=\underset{j}{\sum }[\varepsilon_{\mathrm{HbO}}(\lambda_{1})\Delta [\mathrm{HbO}{]}_{j}+\varepsilon_{\mathrm{HbR}}(\lambda_{1})\Delta [\mathrm{HbR}{]}_{j}]\cdot L_{j}(\lambda_{1},\rho ).$$ (7)

$$A(\lambda_{2},\rho )=\underset{j}{\sum }[\varepsilon_{\mathrm{HbO}}(\lambda_{2})\Delta [\mathrm{HbO}{]}_{j}+\varepsilon_{\mathrm{HbR}}(\lambda_{2})\Delta [\mathrm{HbR}{]}_{j}]\cdot L_{j}(\lambda_{2},\rho ).$$ (8)

and (b) the use of one source-detector separation for each layer in which concentration changes of HbO or HbR take place (i.e., if concentration changes need to be determined in, say, three different layers, then three distinct source-detector separations must be considered for the system to be solvable). Written in matrix form for just two wavelengths

$$[\begin{array}{cccc} A(\lambda_{1},\rho_{1}) & A(\lambda_{1},\rho_{2}) & \ldots & A(\lambda_{1},\rho_{N})\\ A(\lambda_{2},\rho_{1}) & A(\lambda_{2},\rho_{2}) & \ldots & A(\lambda_{2},\rho_{N}) \end{array}]=[\begin{array}{cc} \varepsilon_{\mathrm{HbO}}(\lambda_{1}) & \varepsilon_{\mathrm{HbR}}(\lambda_{1})\\ \varepsilon_{\mathrm{HbO}}(\lambda_{2}) & \varepsilon_{\mathrm{HbR}}(\lambda_{2}) \end{array}][\begin{array}{ccc} \Delta [\mathrm{HbO}{]}_{1} & \ldots & \Delta [\mathrm{HbO}{]}_{N}\\ \Delta [\mathrm{HbR}{]}_{1} & \ldots & \Delta [\mathrm{HbR}{]}_{N} \end{array}][\begin{array}{ccc} L_{1}(\rho_{1}) & \ldots & L_{1}(\rho_{N})\\ \vdots & \ddots & \vdots \\ L_{N}(\rho_{1}) & \ldots & L_{N}(\rho_{N}) \end{array}].$$ (9)

If more chromophores are to be determined, more wavelengths must be considered (however, that is not the scope of this work). Here, two points must be stressed out: first, the A matrix [left hand side of Eq. (9)] evolves with time as well as the Δ[HbX] matrix, so this equation holds for every timepoint of the measurement. Second, the full system, i.e., the one that considers changes in attenuation and chromophore concentration changes in all the N layers of the medium, is often unnecessary, since the metabolic changes in the human head occur mainly in the scalp and cortical layers, while the others remain unaffected. This situation leads to a convenient yet rigorous simplification of the system (9), that can now be rewritten as follows:

$$[\begin{array}{cc} A(\lambda_{1},\rho_{1}) & A(\lambda_{1},\rho_{2})\\ A(\lambda_{2},\rho_{1}) & A(\lambda_{2},\rho_{2}) \end{array}]=[\begin{array}{cc} \varepsilon_{\mathrm{HbO}}(\lambda_{1}) & \varepsilon_{\mathrm{HbR}}(\lambda_{1})\\ \varepsilon_{\mathrm{HbO}}(\lambda_{2}) & \varepsilon_{\mathrm{HbR}}(\lambda_{2}) \end{array}][\begin{array}{cc} \Delta [\mathrm{HbO}{]}_{U} & \Delta [\mathrm{HbO}{]}_{L}\\ \Delta [\mathrm{HbR}{]}_{U} & \Delta [\mathrm{HbR}{]}_{L} \end{array}][\begin{array}{cc} L_{U}(\rho_{1}) & L_{U}(\rho_{2})\\ L_{L}(\rho_{1}) & L_{L}(\rho_{2}) \end{array}].$$ (10)

The subscripts U and L stand for upper and lower, respectively. This convention generalizes the usage of models with different number of layers, regarding that only in two of them the metabolic changes take place. It is important to recall that although head models with more than two layers are considered, the number of detectors and, in turn, the number of equations needed, will be always equal to the number of layers in which metabolic changes occur.

## 3 Data Generation and Reconstruction Approaches

### 3.1 Monte Carlo Simulations

**Fig. 2:**
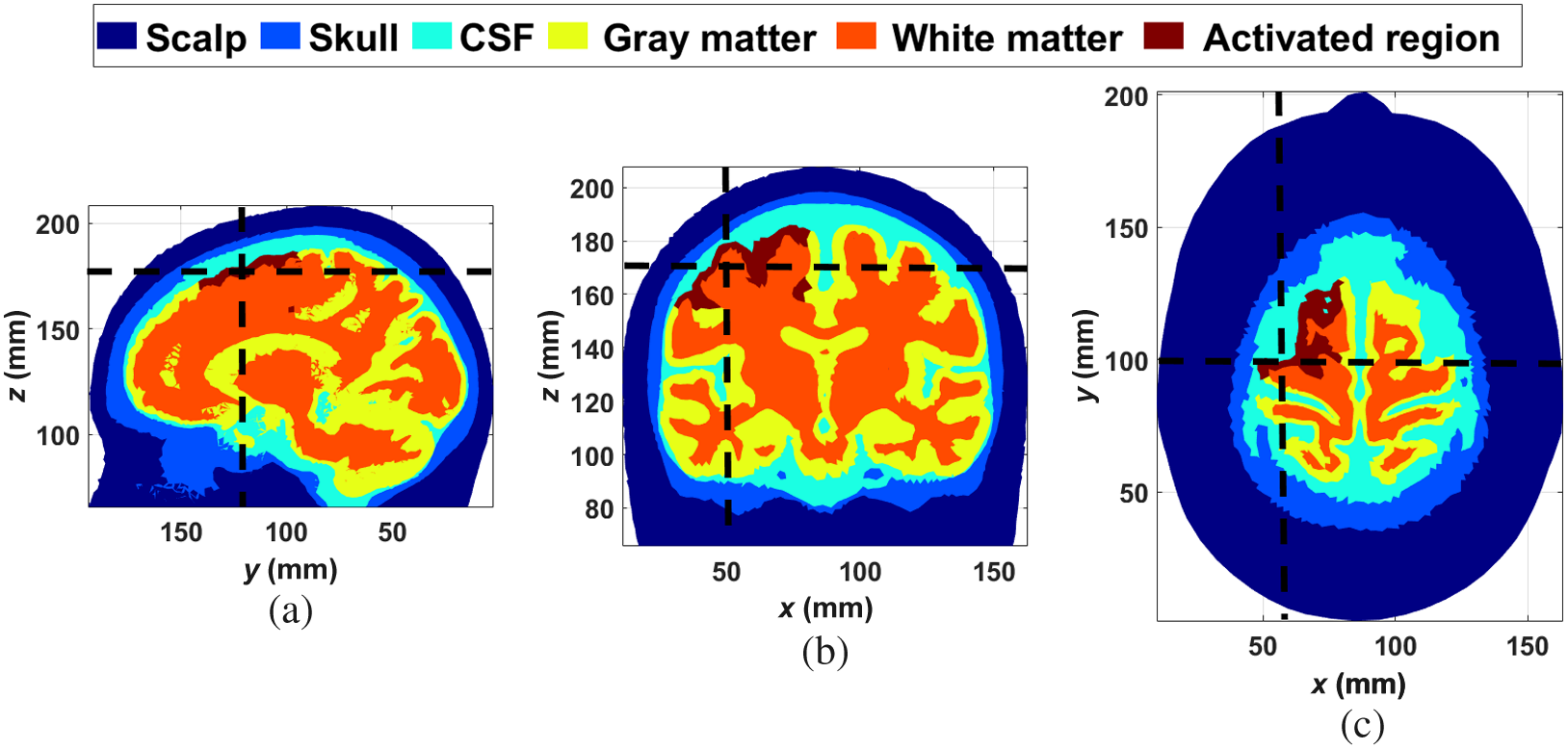
Model of the human head used in the MC simulations. (a) Sagittal slice, (b) coronal slice, and (c) axial slice. The dark red zone represents the activated region. In panel (c), the slice was taken at an appropriate depth to better appreciate the active region. The dashed lines in each subplot indicate the position of the cuts in the complementary subplots.

As it is well known, MC simulations represent the gold standard for light propagation in turbid media, especially when it is not possible to find suitable analytical solutions to this problem; this is the case of photons propagating inside arbitrary geometries, for example, a real human head model. In this work, MC simulations were performed in a five-layered human head model obtained from the Brain2mesh project.^35^ The medium represents the segmented head of a 44-year-old male. As it is a common practice in NIRS and fNIRS,^39^^,^^40^ the five layers considered are scalp (SC), skull (SK), cerebrospinal fluid (CSF), gray matter (GM), and white matter (WM). Figure 2 shows the medium considered, with the layers (and the active region) shown in different colors.

**Fig. 3:**
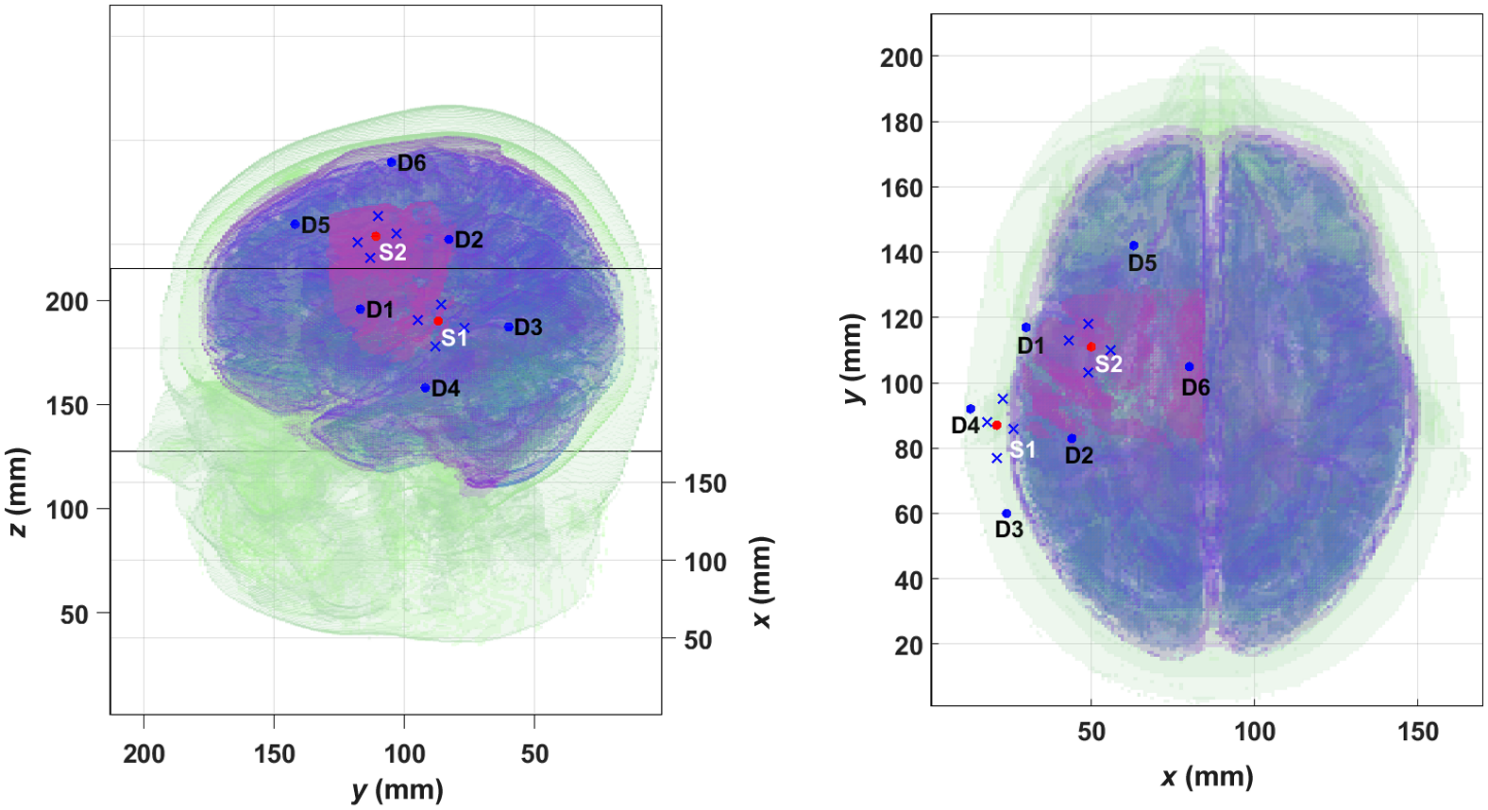
Scheme of measurement optodes in the real human head model. The blue crosses represent the location of the short-separation detectors, while the blue dots show the location of the long-separation detectors (D1–D6); the red dots (S1 and S2) indicate the Gaussian sources. The magenta patch represents the activated area. At left, perspective view, and at right, the top view. Source S1 is linked to long-separation detectors D1–D4 and source S2 is linked to long-separation detectors D1, D2, D5, and D6. Every long channel has a short channel associated with it. See Table 2 for detailed information.

In order to simulate activation processes, variations in chromophore concentrations, Δ[HbX], over baseline concentrations, $[\mathrm{HbX}{]}_{i}$, were proposed. Physiologically, chromophore concentration changes, and therefore absorption variations, take place in laterally limited regions of the cortex related to the elicited stimuli received or tasks performed by the subject. However, to the best of our knowledge, changes in chromophore concentrations in the scalp are not necessarily limited to certain regions and can happen in all its extensions.^19^^,^^41^^–^^43^ To emulate this situation as rigorously as possible, every change in the scalp’s absorption coefficient took place in all layers, while the absorption change that corresponds to the cortical activation was emulated in a portion of the gray matter, segmented for such a goal. This region was created by intersecting every voxel labeled as 4 (which corresponds to gray matter, the fourth layer from outside) with a cylinder, the axis of which, normal to the sagittal plane, passes exactly by the location of source S1 (see Fig. 3 and Table 2), and has a radius of 30 mm.

Regarding the MC simulations details, the so called split-voxel MC program was used;^44^ it allows smoothing of the internal and external contours of a volume, which is especially useful when working with geometries with irregular interfaces as in the human head. This tool is part of the MCX project,^44^^–^^47^ a toolkit that offers the possibility of performing MC simulations of light propagation under different modalities, being the CUDA-parallelized mode the chosen one for this work.

All the simulations were run under the MATLAB environment, on a workstation with an 8-core AMD Ryzen 7 5700X, with RAM of 64 GB and a Nvidia Titan XP GPU. A total of 2000 simulations, consisting of launching $10^{8}$ photons, were run for each situation considered (see the next section), and each one took an average time of 35 s.

The initial basal absorption and scattering coefficients were taken mainly from Okada et al., 1995^38^ and Fang et al., 2010,^47^ for λ=800  nm. Table 1 summarizes all the optical properties. Note that, in this case, the optical properties chosen for the CSF favor a diffusive regime for light propagation, something that is not strictly true in real situations;^38^^,^^48^^,^^49^ however, this choice is justified by the fact that we are comparing MC reconstructions to analytical reconstructions derived from the diffusive approximation.

**Table 1:** Summary of each layer’s absorption coefficient, scattering coefficient, anisotropy factor (g), and refractive index (n), together with the mean thickness (d) at the plane defined by the optodes, parallel to the XY plane.

—	$\mu_{a}$ ${(\mathrm{mm}}^{-1})$	$\mu_{s}$ ${(\mathrm{mm}}^{-1})$	g	n	d (mm)
SC	0.018	19	0.9	1.33	10
SK	0.016	16	0.9	1.33	3
CSF	0.002	10	0.9	1.33	3
GM	0.036	22	0.9	1.33	2
WM	0.014	41	0.9	1.33	—

The absorption coefficient of the first (upper) and fourth (lower) layers, i.e., scalp and gray matter, where converted to basal HbO and HbR concentrations for $\lambda_{1}=690\;\mathrm{nm}$ and $\lambda_{2}=830\;\mathrm{nm}$, by solving the system

$$[\begin{array}{cc} \Delta [\mathrm{HbO}{]}_{U} & \Delta [\mathrm{HbO}{]}_{L}\\ \Delta [\mathrm{HbR}{]}_{U} & \Delta [\mathrm{HbR}{]}_{L} \end{array}]={\left[\begin{array}{cc} \varepsilon_{\mathrm{HbO}}(690) & \varepsilon_{\mathrm{HbR}}(690)\\ \varepsilon_{\mathrm{HbO}}(830) & \varepsilon_{\mathrm{HbR}}(830) \end{array}\right]}^{-1}[\begin{array}{cc} \Delta \mu_{a,U}(800) & \Delta \mu_{a,L}(800)\\ \Delta \mu_{a,U}(800) & \Delta \mu_{a,L}(800) \end{array}],$$ (11)

this conversion from absorption at 800 nm to HbX concentrations at 690 and 830 nm, although not so rigorous, relies solely on the need to have an estimation of the reference values at the beginning of the measurement process. Then, both the baseline and the corresponding concentration changes were converted into absorption coefficients for the two different wavelengths, $\lambda_{1}=690\;\mathrm{nm}$ and $\lambda_{2}=830\;\mathrm{nm}$, by the following operation:

$$[\begin{array}{cc} \Delta \mu_{a,U}(690) & \Delta \mu_{a,L}(690)\\ \Delta \mu_{a,U}(830) & \Delta \mu_{a,L}(830) \end{array}]=[\begin{array}{cc} \varepsilon_{\mathrm{HbO}}(690) & \varepsilon_{\mathrm{HbR}}(690)\\ \varepsilon_{\mathrm{HbO}}(830) & \varepsilon_{\mathrm{HbR}}(830) \end{array}][\begin{array}{cc} \Delta [\mathrm{HbO}{]}_{U} & \Delta [\mathrm{HbO}{]}_{L}\\ \Delta [\mathrm{HbR}{]}_{U} & \Delta [\mathrm{HbR}{]}_{L} \end{array}].$$ (12)

For each simulation, the initial and final absorption coefficients elicited by the corresponding chromophores concentration changes were combined in time blocks. To emulate a more realistic situation involving humans, each block was convolved with a canonical hemodynamic response function (HRF).^50^

The MC simulations were then fed with these blocks of temporal evolution of $\mu_{a}$ for each wavelength. The measurement scheme was modeled as follows: two Gaussian sources impinged normally to the scalp surface, and for each source, eight detectors (each of them having a radius of 2 mm) were placed. These are divided into four long-separation detectors and four short-separation detectors. They were placed around the sources and over the primary motor cortex and the supplementary motor area, on the left hemisphere, being both regions commonly associated with right limb mobility.^9^^,^^51^^,^^52^ Table 2 shows the details of this layout.

**Table 2:** Summary of the information regarding the layout schemed in Fig. 3. Column 3 lists the number corresponding solely to the long channels. Column 4 shows the distance between each source and (i) the long-separation detector (without parentheses); and (ii) the short-separation detector (in parenthesis). In column 5, the empty cells indicate that the corresponding channel does not fully lie over the active region.

Source	Detector	Long channel No.	Separation (mm)	Over the activated region?
1	1	1	31.3 (8.3)	Yes
1	2	2	32.1 (6.5)	
1	3	3	27.6 (10.4)	No
1	4	4	27.7 (10.5)	No
2	5	5	33.9 (7.3)	
2	6	6	33.2 (7.9)	Yes
2	2	7	28.8 (8.3)	Yes
2	3	8	28.9 (8.9)	Yes

Then, considering each pair formed by a source and a long-separation detector (with the corresponding short-separation detector), together with the two wavelengths mentioned above, the resulting datasets had dimensions of T×2×2, where T is the total number of timepoints of each block. Figure 3 is a scheme of the whole head where the short-separation detectors are shown as blue crosses, the long-channel detectors as blue dots and the sources are represented by red dots.

### 3.2 Concentration Changes Proposals

We have considered two hypothetical situations, which are opposite from each other. This choice is intended to summarize the wide spectrum of possibilities arising from the combination of hemodynamic activities in both the scalp and the cortex when considering real clinical cases;^16^^,^^18^^,^^53^^–^^57^ hence, we have assumed that any of these combinations could lie within the situations depicted in this work.

• Situation 1: the time series begins with a resting state of 8 s, followed by a period of activation of 8 s that consists of: (1) an increase of 10% in $[\mathrm{HbO}{]}_{U}$ and a decrease of 5% in $[\mathrm{HbR}{]}_{U}$, and (2) an increase of 50% in $[\mathrm{HbO}{]}_{L}$ and a decrease of 25% in $[\mathrm{HbR}{]}_{L}$. Both concentration changes occur at the same time. A final resting state of 32 s finishes each block.
• Situation 2: now the time series begins with a resting state period of 6 s. Then, a decrease of 25% in $[\mathrm{HbO}{]}_{U}$ of 8 s occurs in the upper layer, while the $[\mathrm{HbR}{]}_{U}$ in this layer remains constant. The $[\mathrm{HbO}{]}_{U}$ returns to its baseline value and remains constant for the last 36 s. Regarding the changes in the lower layer, the block starts with a 10 s resting state; after that the activation of the lower layer is produced, and it consists of an increase in $[\mathrm{HbO}{]}_{L}$ of 50% and a decrease of 25% in $[\mathrm{HbR}{]}_{L}$; it has a total duration of 12 s. A resting period of 28 s occurs at the end of the activation period.

**Fig. 4:**
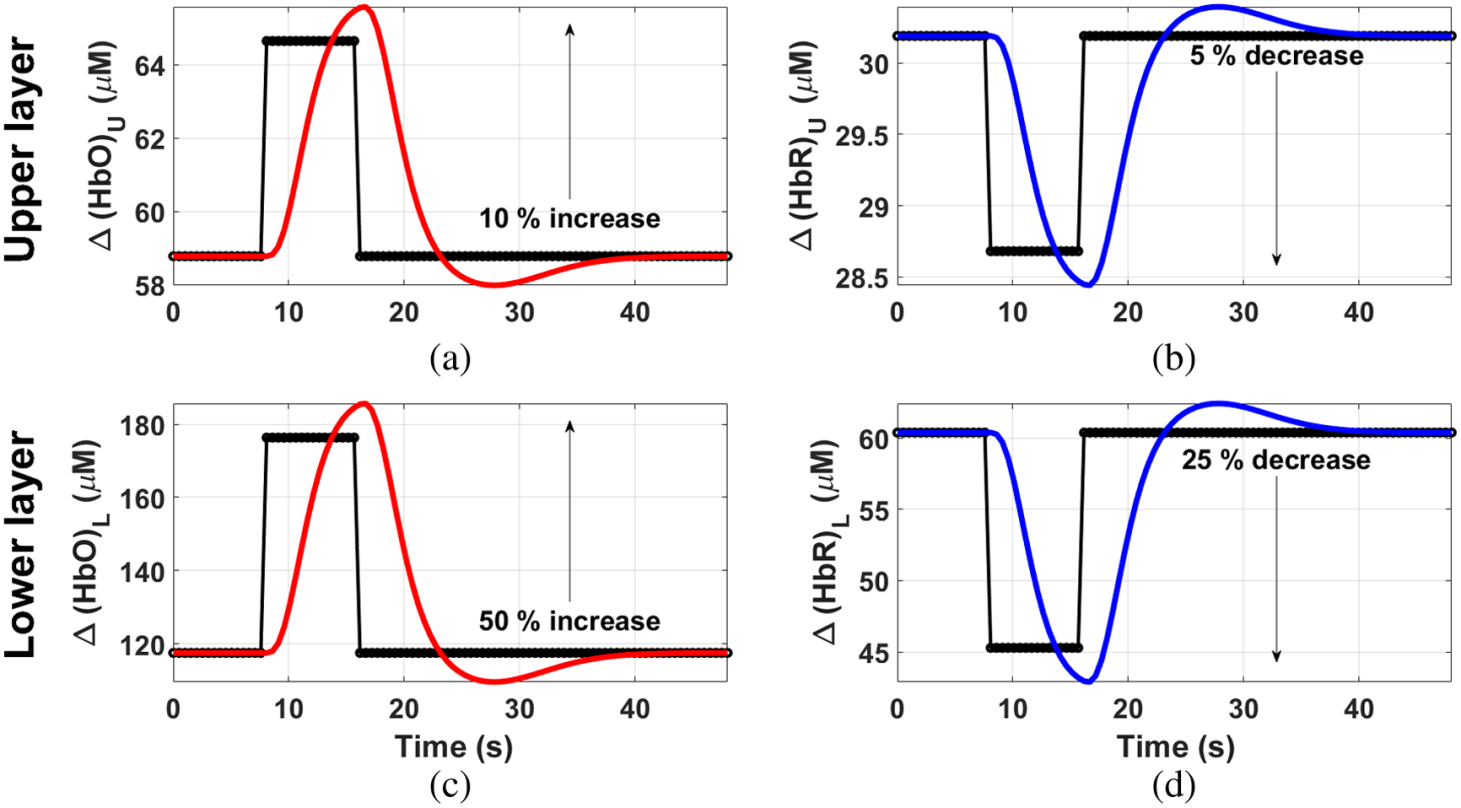
Scheme of a complete block of changes in [HbO] in the upper (a) and lower (c) layers; and [HbR] in the upper (b) and lower (d) layers for situation 1. Black dotted lines: before convolving with the HRF. Colored lines: after the convolution with the HRF.

**Fig. 5:**
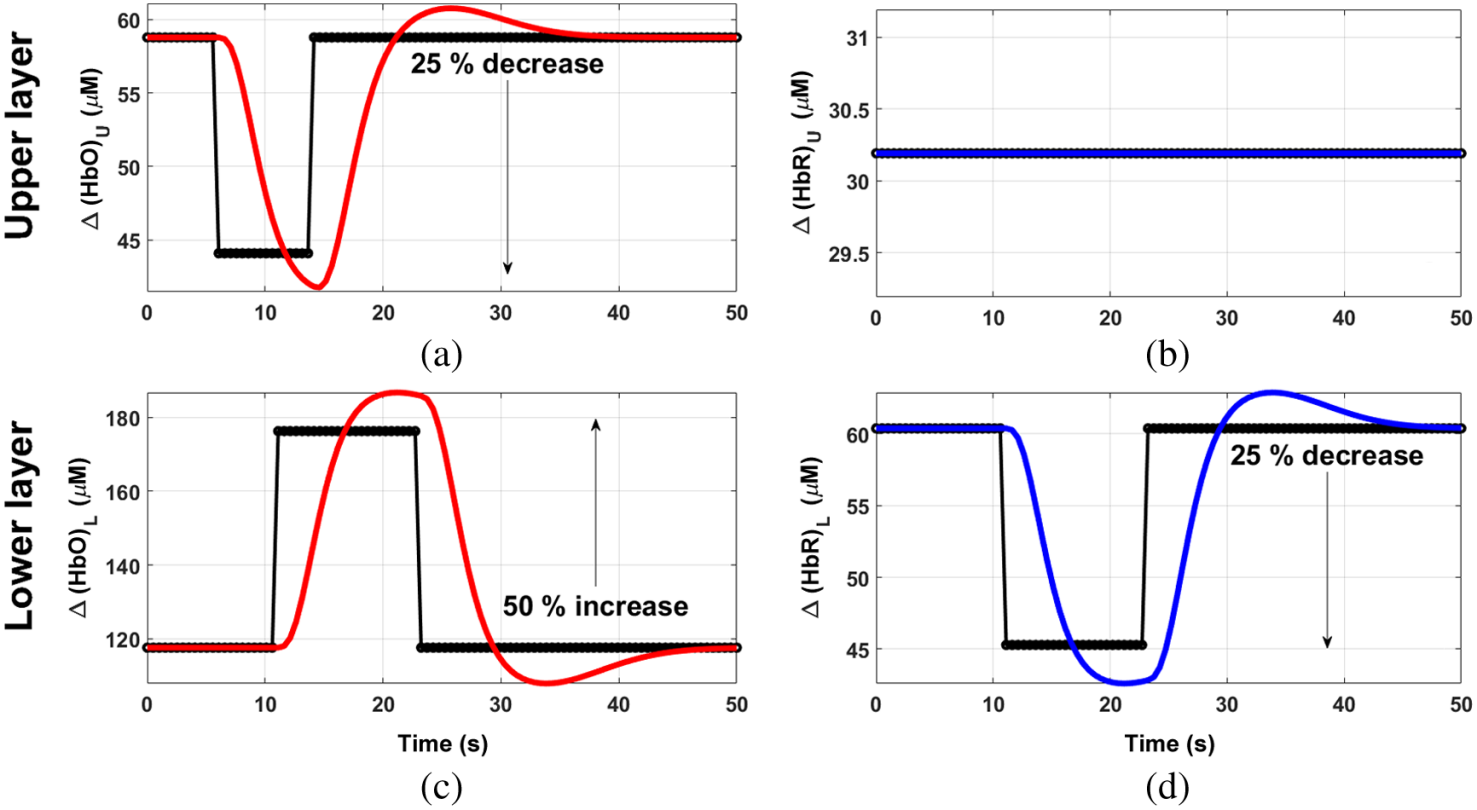
Scheme of a complete block of changes in [HbO] in the upper (a) and lower (c) layers; and [HbR] in the upper (b) and lower (d) layers for situation 2. Black dotted lines: before convolving with the HRF. Colored lines: after the convolution with the HRF.

These two situations are respectively depicted in Figs. 4 and 5 (together with the corresponding HRF convolutions, as stated in the previous section), while Table 3 shows a summary of the initial and final states of both, absorption coefficients and chromophore concentrations.

**Table 3:** Summary of initial and final absorption coefficients (columns 3 and 4) and the corresponding initial and final chromophores concentrations (columns 5 and 6) for situations 1 and 2. In columns 4, 5, and 6, the first element of each square bracket represents the corresponding value for $\lambda_{1}=690\;\mathrm{nm}$ and the second one, for $\lambda_{2}=830\;\mathrm{nm}$. Note that the initial absorption coefficients correspond to $\lambda_{\text{isosbestic}}=800\;\mathrm{nm}$.

Situation	Layer	$\mu_{a,i}$ (${\mathrm{mm}}^{-1}$)	$\mu_{a,f}$ (${\mathrm{mm}}^{-1}$)	$[\mathrm{HbX}{]}_{i}$ (μM)	$[\mathrm{HbX}{]}_{f}$ (μM)
1	U	(0.018)	(0.0183;0.0169)	(58.78;30.19)	(64.66;28.68)
	L	(0.036)	(0.0326;0.0468)	(117.55;60.38)	(176.33;45.29)
2	U	(0.018)	(0.0188;0.0153)	(58.78;30.19)	(44.08;30.19)
	L	(0.036)	(0.0326;0.0468)	(117.55;60.38)	(176.33;45.29)

A real fNIRS measurement is usually arranged in a block structure consisting of several repetitions of resting and activity periods. To emulate this situation, each block described above was replicated five times, achieving in this way time series of 48  s×5=240  s (for situation 1) and 50  s×5=250  s (for situation 2).

### 3.3 Retrieval of Concentration Changes

Several reconstruction approaches were considered. On the one hand, a homogeneous retrieval was performed as it is presently the most-used method, and it is therefore the one to compare with. On the other hand, two- and five-layered retrievals were performed using analytical MPPLs. Additionally, MC-based reconstructions were also done and used as a reference.

#### 3.3.1 Homogeneous reconstruction

In the particular case of the homogeneous reconstruction, it is a common practice to replace the exact MPPLs of photons in the medium by a factor that takes into account the scattering of the medium: the differential pathlength factor, DPF^9^^,^^58^. Equation (6) can now be stated as

$$A(\lambda ,\rho )=\{(\varepsilon_{\mathrm{HbO}}(\lambda )\cdot \Delta [\mathrm{HbO}]+\varepsilon_{\mathrm{HbR}}(\lambda )\cdot \Delta [\mathrm{HbR}])\}\cdot \mathrm{DPF}\cdot \rho .$$ (13)

Then, Eq. (9) takes the form

$$[\begin{array}{c} A(\lambda_{1},\rho )\\ A(\lambda_{2},\rho ) \end{array}]=[\begin{array}{cc} \varepsilon_{\mathrm{HbO}}(\lambda_{1}) & \varepsilon_{\mathrm{HbR}}(\lambda_{1})\\ \varepsilon_{\mathrm{HbO}}(\lambda_{2}) & \varepsilon_{\mathrm{HbR}}(\lambda_{2}) \end{array}][\begin{array}{c} \Delta [\mathrm{HbO}]\\ \Delta [\mathrm{HbR}] \end{array}]\cdot \mathrm{DPF}\cdot \rho .$$ (14)

For this case, the solution to Eq. (14) is straightforward

$$[\begin{array}{c} \Delta [\mathrm{HbO}]\\ \Delta [\mathrm{HbR}] \end{array}]={\left[\begin{array}{cc} \varepsilon_{\mathrm{HbO}}(\lambda_{1}) & \varepsilon_{\mathrm{HbR}}(\lambda_{1})\\ \varepsilon_{\mathrm{HbO}}(\lambda_{2}) & \varepsilon_{\mathrm{HbR}}(\lambda_{2}) \end{array}\right]}^{-1}[\begin{array}{c} A(\lambda_{1},\rho )\\ A(\lambda_{2},\rho ) \end{array}]\cdot \frac{1}{\mathrm{DPF}\cdot \rho },$$ (15)

where we have considered DPF = 6.^21^^,^^50^^,^^59^ The scalp influence can be reduced by correcting A(λ,ρ) as proposed by Saager et al.;^18^ this approach requires the use of short channels together with the conventional long channels

$$A{\left(\lambda_{j},\rho_{2}\right)}^{\prime }=A(\lambda_{j},\rho_{2})-K\cdot A(\lambda_{j},\rho_{1}),$$ (16)

$$K=\frac{\left\langle A(\lambda_{j},\rho_{1}),A(\lambda_{j},\rho_{2})\right\rangle }{\left\langle A(\lambda_{j},\rho_{1}),A(\lambda_{j},\rho_{1})\right\rangle },$$ (17)

where the brackets indicate inner product (and it is assumed that $\rho_{1}<\rho_{2}$). It must be pointed out that if the changes taking place in each layer are temporally correlated, i.e., they occur at the same time and have the same duration, the K factor is zero, so no correction is done. Alternatively, another common way to take into account the objective region where the metabolic changes occur is the use of the partial pathlength factor.^22^^,^^23^ This factor has a value between 0.09 and 0.11 for wavelengths of 690 to 830 nm.^60^

#### 3.3.2 Two- and five-layered reconstructions

To retrieve Δ[HbO] and Δ[HbR] coming from both the scalp and the cortex, two- and five-layered models were used. In both cases, a singular value decomposition approach was implemented. Regarding the two-layered model, we treated the attenuation data as if they had been generated by a two-layered medium formed by a cerebral layer plus an extracranial layer. To do so, the MPPLs of a two-layered medium were obtained using the analytical model,^33^^,^^34^ taking the scalp as the extracranial layer, and merging the skull, CSF, gray matter, and white matter to form the lower semi-infinite layer.

With respect to the five-layered reconstruction, the real segmented head anatomy was considered. To obtain the MPPLs, again, the analytical approach was adopted, in an analogous way to the one mentioned before for the two-layered medium. Now, the (mean) thickness of each layer was contemplated. Please recall that considering a five-layered model does not add any difficulty to the retrieval computations, since there are only two layers in which the metabolic changes occur.

As a last remark, the thicknesses of the scalp and skull layers were obtained using MATLAB-assisted tools, such as Imtool.

## 4 Results

We begin this section by comparing the different reconstruction approaches for one particular channel (channel #1, fully placed on the active region of the cortex) to determine which model performs the best under the situations introduced in Sec. 3.2. Once this choice is established, we make a general comparison for all channels between this model and the homogeneous one. We decided to proceed in this way to avoid an excessive number of figures in the manuscript. However, the remaining results are available under request for the interested reader. Once these comparisons are done, we discuss in particular how the chosen analytical reconstruction depends on the initial optical parameters and the thicknesses of the layers considered in this work. Finally, we perform an analysis of the computational costs involved in retrieving the metabolic changes with the different reconstruction methods proposed here.

### 4.1 Reconstruction Results for Channel #1

#### 4.1.1 Situation 1

**Fig. 6:**
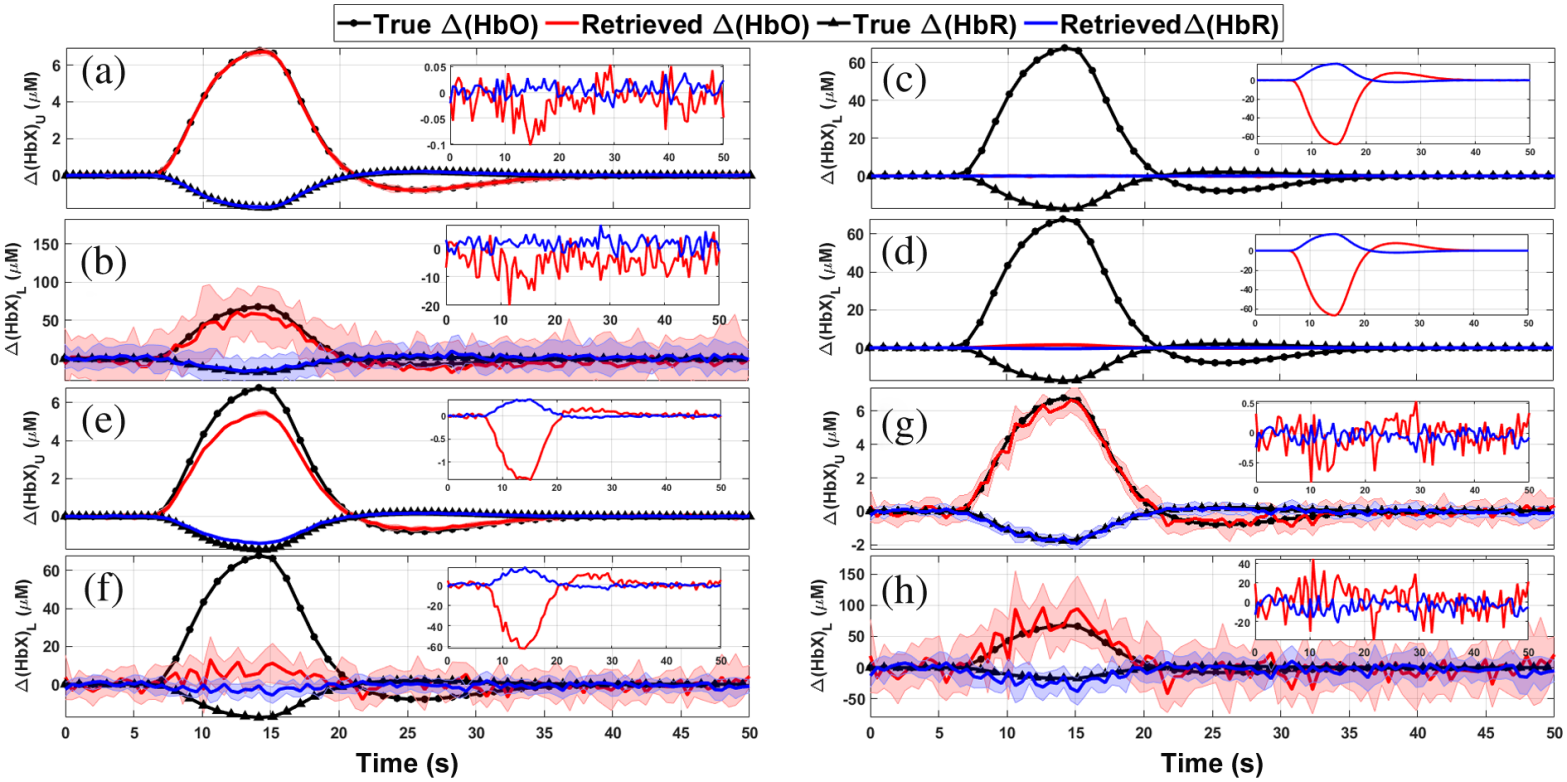
Retrievals computed with four different approaches for channel #1 in situation 1 (the shaded regions represent one standard deviation from mean value). In panels (a) and (b), using the five-layered model with the MPPLs obtained by MC, for both, upper and lower layers. In panels (c) and (d), homogeneous retrieval only for the lower layer using the corrected signal and the DPF approach, respectively. In panels (e) and (f), the reconstructions performed with the two-layered model, and in panels (g) and (h), the retrieval obtained with the five-layered model. For the last two cases, the MPPLs were obtained analytically. In each inset, the corresponding residuals are shown.

Figures 6(a) and 6(b) show the reconstruction of Δ[HbX] for both the upper (scalp) and lower (cortex) layers obtained using the MC-based MPPLs. As it can be seen, the matching between the retrieval and the proposal is almost perfect. It can also be noted that the difference between the reconstructions and the true chromophore concentration changes, as well as the uncertainties associated to the retrievals (represented by the shaded areas in the plots), are much smaller for the upper layer than for the lower layer. In the case of the upper layer, this can be explained by the fact that most of the detected photons travel through the upper layer, thus improving the statistics of the retrieved HbO and HbR signals; on the other hand, a rather small proportion of the detected photons explore the deeper region, which translates into poorer statistics of the corresponding HbO and HbR signals.

In summary, the goodness of the MC-based reconstruction just shown above justifies its use as a reference.

Figures 6(c) and 6(d) show the retrieved chromophore concentration changes by means of the homogeneous model for both, the corrected and uncorrected attenuations (see Sec. 3.3.1). The reason why the corrected signal retrieval returns just flat lines is because the signals in both the short and the long channels are temporally correlated. The uncorrected reconstruction, on the other hand, qualitatively matches the proposed changes; however, it largely underestimates the quantification of chromophores concentration changes. This can be due to the fact that the homogeneous model implicitly considers that these changes take place in a volume that is much bigger than the real one, thus compensating the Δ[HbX] retrievals.

Retrievals by means of the two-layered model using analytical MPPLs are shown in Figs. 6(e) and 6(f). The quality of the reconstruction outperforms that of the homogeneous retrieval, although the results are far from the proposed ones (relative differences of up to 30% for $\Delta [\mathrm{HbO}{]}_{U}$ and 65% for $\Delta [\mathrm{HbO}{]}_{L}$, and 25% for $\Delta [\mathrm{HbR}{]}_{U}$ and 50% $\Delta [\mathrm{HbR}{]}_{L}$).

The five-layered reconstruction using the analytical MPPLs is shown in Figs. 6(g) and 6(h). The retrieval for the fourth layer is in good agreement with the proposals, being the largest relative differences less than 25% for both Δ[HbO] and Δ[HbR]. The recovery for the upper layer outperforms the one obtained by the two-layered analytical model, something reasonable given the specificity of the model in terms of the number of layers.

Comparing these last sets of results (the two-layered and the five-layered analytical methods) with the MC reconstruction, it can be easily observed that the five-layered analytical model is the closest one to the MC goal results. The failure of the two-layered model can be attributed to the way in which the layers in the head are merged (the upper layer for the scalp and the lower layer for the remaining tissue); probably a different choice would have led to better results in the lower layer, but increasing the error in the estimations for the upper layer.

#### 4.1.2 Situation 2

**Fig. 7:**
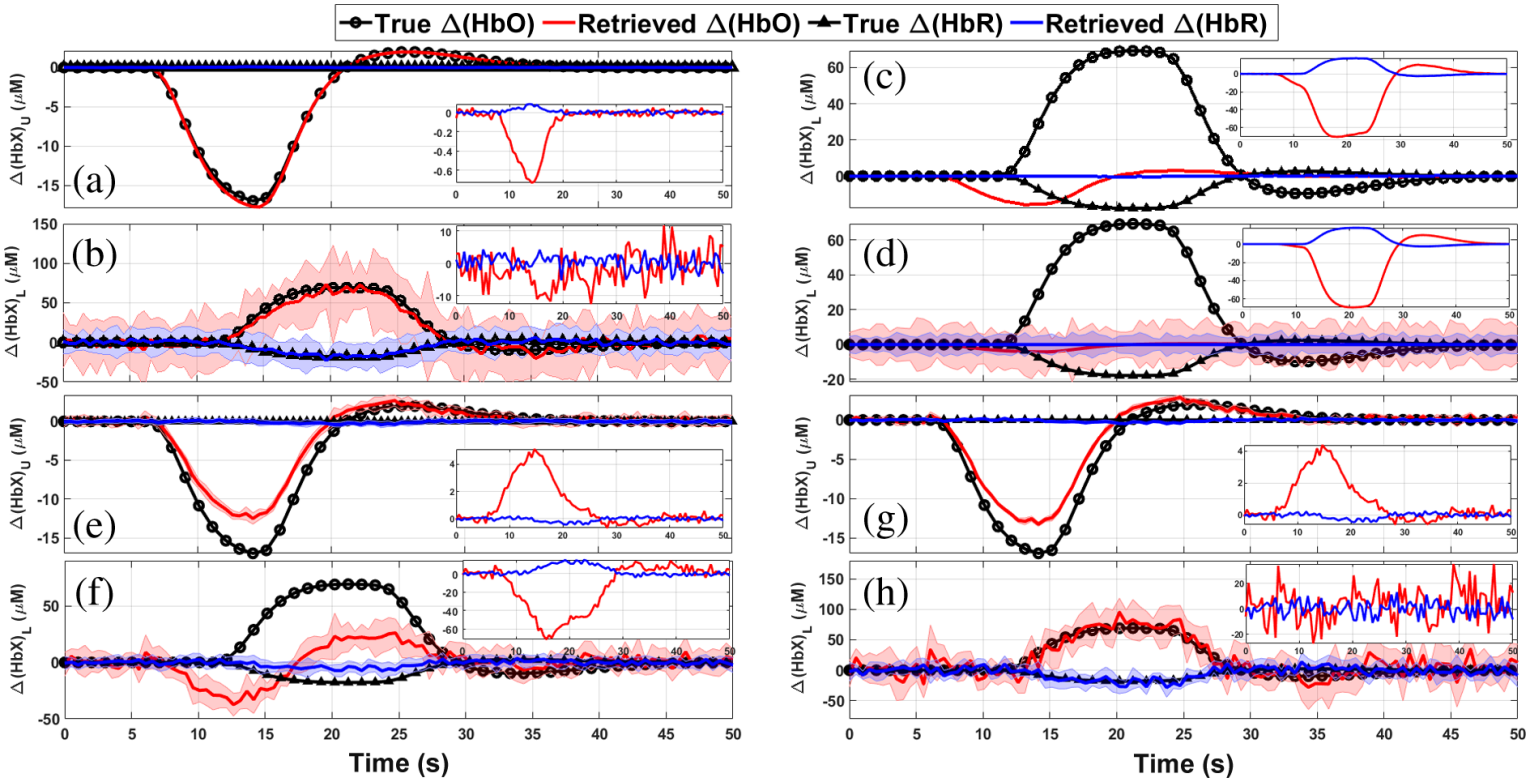
Retrievals computed with four different approaches for channel #1 in situation 2 (the shaded regions represent one standard deviation from mean value). In panels (a) and (b), using the five-layered model with the MPPLs obtained by MC, for both, upper and lower layers. In panels (c) and (d), homogeneous retrieval only for the lower layer using the corrected signal and the DPF approach, respectively. In panels (e) and (f), the reconstructions performed with the two-layered model, and in panels (g) and (h), the retrieval obtained with the five-layered model. For the last two cases, the MPPLs were obtained analytically. In each inset, the corresponding residuals are shown.

Figures 7(a) and 7(b) show the reconstruction of Δ[HbX] for both the upper (scalp) and lower (cortex) layers, respectively, obtained using the MC-based MPPLs, now for situation 2. Again, the retrieval is in excellent agreement with the proposal, even considering the timing mismatch (shifts and durations) and the inverted sign of Δ[HbO] between the activities in both layers.

Figures 7(c) and 7(d) show the corrected (upper plots) and the uncorrected (lower plots) homogeneous reconstructions together with the corresponding residuals. Although situation 2 was intended to generate low-correlated data between layers (being the correlation coefficient between the proposed $\Delta [\mathrm{HbO}{]}_{U}$ and $\Delta [\mathrm{HbO}{]}_{L}∼-0.18$), the attenuation for both the long and the short channels resulted in a correlation of ∼0.87. This is why the corrected reconstruction is so poor when compared with the proposed concentration changes in the cortex tissue. On the other hand, the lack of correction not only leads to an underestimation of the $\Delta [\mathrm{HbO}{]}_{L}$ change, but it also introduces an opposite sign (often named “inverse response” in the literature),^61^^–^^63^ coming without doubt from the changes of oxyhemoglobin in the scalp tissue. In the end, none of these approaches appropriately retrieves what is really going on in the medium.

The reconstruction performed with the two-layered analytical model is depicted in Figs. 7(e) and 7(f). The quality of the chromophores changes retrieval is similar to the one given by situation 1, i.e., the changes in both layers is underestimated; however, we must consider once again that situation 2 is characterized by a time shift in the activation in both layers, as well as different activity durations together with changes of opposite signs, something that is appropriately retrieved by the analytical model.

Finally, Figs. 7(g) and 7(h) show the retrieval of Δ[HbO] and Δ[HbR] for the scalp and the cortex using the five-layered theoretical reconstruction. When comparing this with the corresponding two-layered results, we notice that the upper layer recovery presents a similar performance (i.e., the $\Delta [\mathrm{HbO}{]}_{U}$ signal is once again underestimated), while the changes in the bottom layer are more accurately retrieved, greatly resembling that one of the MC reconstruction. Undeniably, the differences between the MC and the five-layered reconstructions for the superficial layer are now more noticeable than in the case of situation 1. Evidently, a relationship between scalp and brain activity signals such as the one given in situation 1 is much more favorable for the analytical five-layered method. For situation 2, on the other hand, the differences between the studied medium and the proposed model (a digital human head versus a stack of optically turbid planar layers) have a stronger impact on the upper layer’s reconstruction. Of course, this is not the case for the corresponding MC results, which makes use of the same digital head model to compute the numerical MPPLs. Besides this, we must recall that the MC simulations are based on the RTE, while our analytical approaches (for both the two- and the five-layered models) rely on the DA.

The discussion given above for both situations leads to the conclusion that the best analytical reconstruction is the one obtained by means of the five-layered model. In the following, we compare this with the most widely extended homogeneous reconstruction, and then we analyze how the goodness of the five-layered reconstruction depends on prior information such as the initial absorption coefficients and the thicknesses of the scalp and cortex layers.

### 4.2 Comparison Between the Homogeneous and the Five-Layered Reconstructions for All Channels

In this section, we study the reconstruction process for the most challenging situation 2 and for all channels, as it is illustrative to see how the homogeneous and the five-layered approaches perform when the different source-detector pairs lie not only over the active region of the brain, but outside as well.

**Fig. 8:**
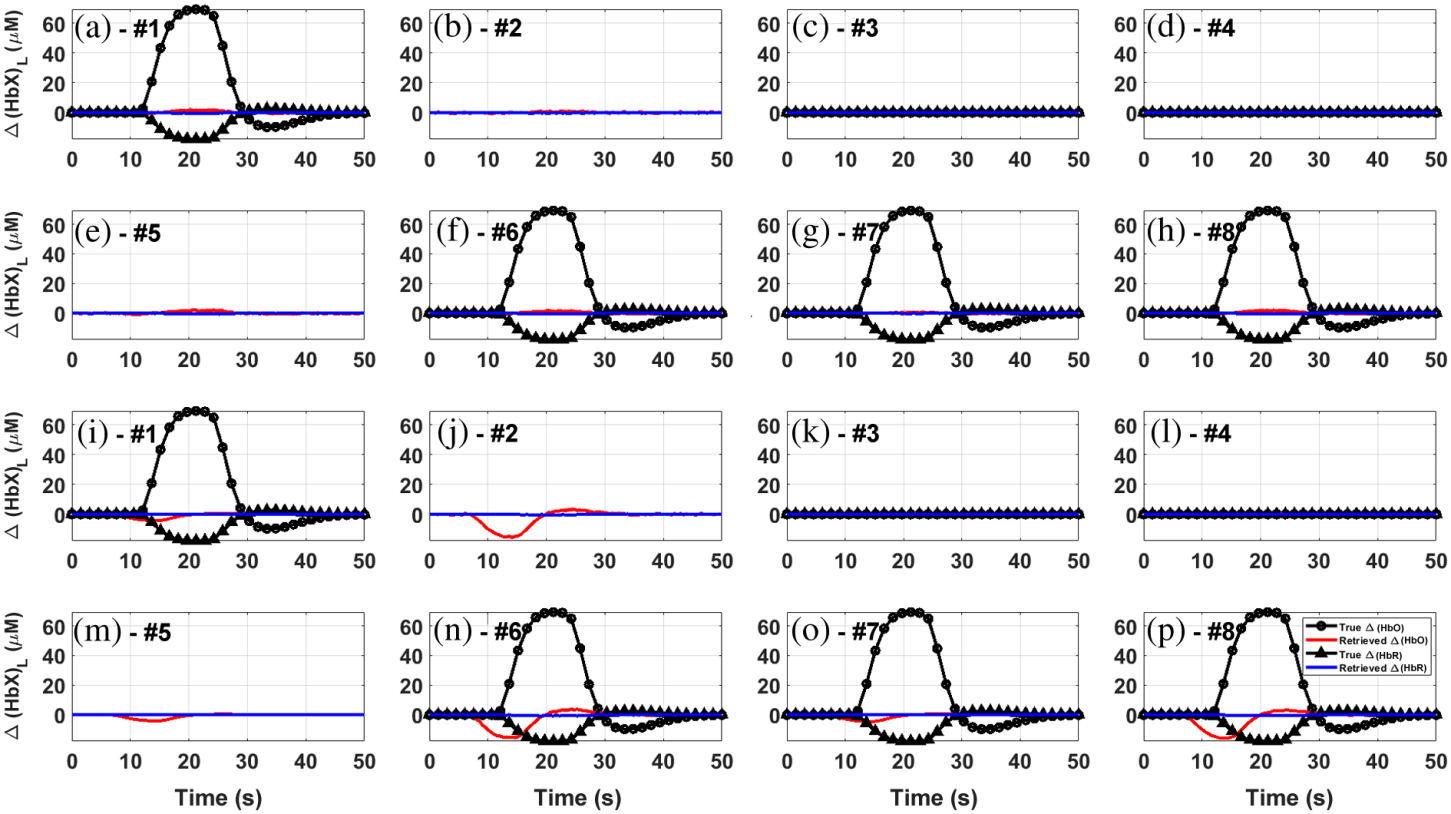
Homogeneous retrieval of chromophore concentration changes in the lower layer using the short-channel-corrected signal [(a)–(h): channels #1 to #8] and the DPF [(i)–(p): channels #1 to #8] approaches, for situation 2 and every channel considered. Shaded regions: one standard deviation from mean value. Flat proposal lines correspond to channels outside of the active region, while absent proposal lines indicate that the corresponding channels do not entirely lie on top of it (please refer to Table 2 and Fig. 3 for further details).

The results for the homogeneous retrieval are summarized in Fig. 8. The first eight subplots [Figs. 8(a)–8(h)] show the retrieved Δ[HbX] values correcting with the short channels, while the next ones [Figs. 8(i)–8(p)] represent the retrievals using only the DPF approach. In all cases, the black lines and symbols indicate the objective behaviors (however, in the case of those channels not completely lying over the active region, no objective curves were added, since it is not clear what the true behaviors should look like). An overall inspection of the whole figure indicates that none of the homogeneous approaches shown here successfully retrieve the proposed concentration changes. On one hand, the short-channels corrected signals seem to detect activation only on the actually active channels, but the retrieved values clearly underestimate the true ones. On the other hand, the DPF approach detects more significant activity; however, the detected activity is inverse and can be seen in all channels (without regarding whether the channel is really active or not), which suggests the presence of a strong contribution from the upper layer.

**Fig. 9:**
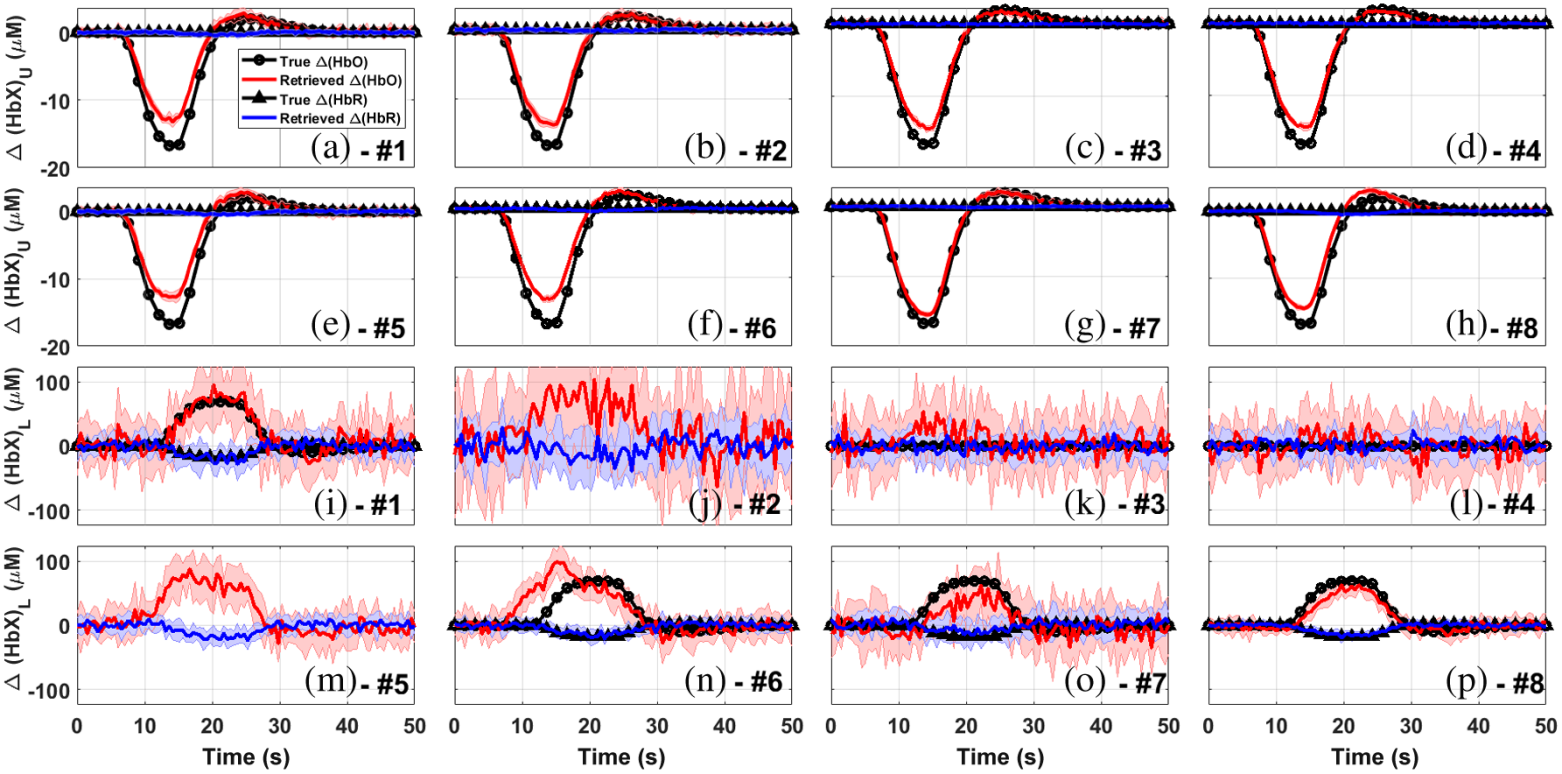
Retrieval of chromophore concentration changes in the upper [(a)–(h): channels #1 to #8] and the lower [(i)–(p): channels #1 to #8] layers, using the analytical-MPPLs-based five-layered approaches, for situation 2 and every channel considered. Shaded regions: one standard deviation from mean value. Flat proposal lines correspond to channels outside of the active region, while absent proposal lines indicate that the corresponding channels do not entirely lie on top of it (please refer to Table 2 and Fig. 3 for further details).

The results for the five-layered reconstruction are shown in Figs. 9(a)–9(h) (for the scalp layer) and in Figs. 9(i)–9(p) (for the cortex layer). The retrieved Δ[HbO] in the upper layer are, in general, smaller in magnitude that the proposed ones, except for channel #7 [Fig. 9(g)], where the agreement is almost perfect. This is probably due to the set of thicknesses (listed in Table 1) used to feed the analytical model (please note that the model needs these values as fixed and exact parameters, but the real thicknesses vary throughout all the region covered by the channel). Regarding the lower layer, the analytical reconstruction seems to be much more robust. Our method detects activity not only where there actually exists, but also with the corresponding sign (positive for HbO and negative for HbR). The influence of the upper layer appears to affect some channels [particularly channels #6 to #8, depicted by Figs. 9(n)–9(p), respectively] in a such a way that the retrieved Δ[HbO] is barely smaller (∼5% and ∼10%) than the proposed changes. Besides, and as expected, no activity is retrieved in channels #3 and #4 [Figs. 9(k) and 9(l)]. As for channels #2 and #5 [corresponding to Figs. 9(j) and 9(m)], the five-layered model detects activation, but here it is difficult to decide whether the channels are truly active or not (particularly for channel #2, given the considerably large error bands for each hemoglobin species) since they do not completely lie on top of the active area.

Overall, the reconstruction based on the analytical five-layered model strongly outperforms the homogeneous reconstruction. Despite that the retrieval in the upper layer is not perfect in quantitative terms, it is still qualitatively correct; most importantly, the HbO and HbR concentration changes retrieved in the cortex layer with our method are much more accurate than their homogeneous counterpart. Additionally, the time shift between the upper and the lower signals are also reproduced with high precision. All this leads to conclude that our method is able to quantitatively reproduce the chromophore concentration changes taking place simultaneously in both the scalp and the cortex tissues, while also predicting the correct behavior (i.e., direct or inverse response) for each chromophore in each layer, something that could probably lead to a reduction in the rate of false positive and negative errors.

### 4.3 Dependence of the Reconstruction on the Use of a Priori Information

As in any retrieving process, the quality of the reconstruction can be improved with an appropriate use of any available *a priori* information. Hence, in real clinical applications this information must be used whenever possible. However, in the case of priors lacking, they must be guessed/inferred. To this end, we studied the root-mean-squared error (RMSE) between the proposals and the retrievals, computed as

$$\mathrm{RMSE}=\sqrt{\frac{1}{n}\overset{n}{\underset{i=1}{\sum }}{\left(\Delta [\mathrm{HbX}{]}_{i,\text{retrieved}}-\Delta [\mathrm{HbX}{]}_{i,\text{proposed}}\right)}^{2}},$$ (18)

where the i index denotes each time channel. We focused on two sets of *a priori* data: the initial absorption coefficients of the objective layers and their corresponding thicknesses. Both studies were performed on the results once again for channel #1 in situation 2, which proved to be more challenging than situation 1.

#### 4.3.1 Dependence on the initial absorption coefficients

The need for initial absorption coefficients involved in the layered reconstructions is manifested through the dependence of the MPPLs with the wavelength [as stated by Eqs. (4) and (5) and subsequent]. It could be claimed that this is a flaw in the method, since the homogeneous retrieval does not require any initial values; however, it must be noted that choosing a specific DPF value implicitly involves the use of an initial absorption coefficient (in this case only one, since we deal with a homogeneous medium).

**Fig. 10:**
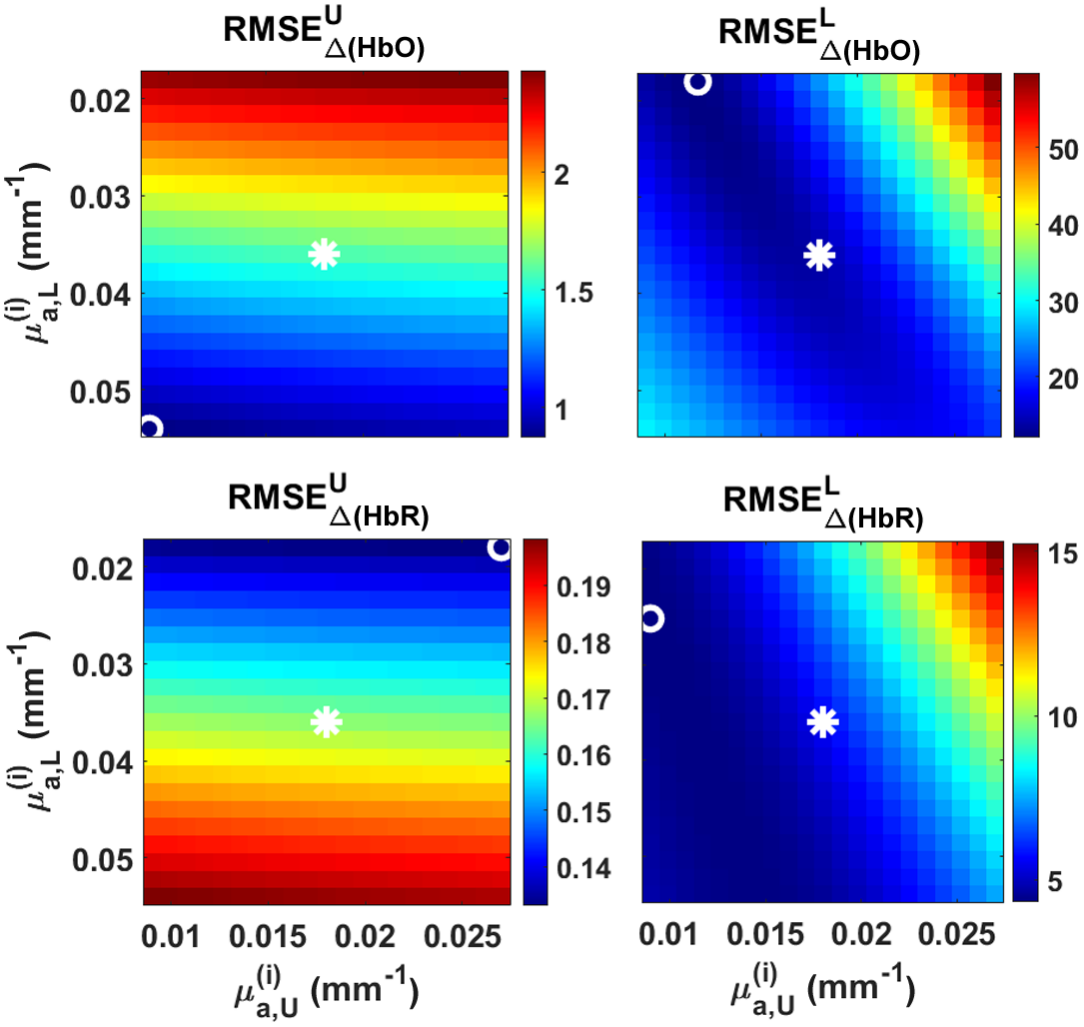
RMSE values obtained by varying the initial absorption coefficients of the upper ($\mu_{a,U}$) and lower ($\mu_{a,L}$) layers (±50% with respect to the true values) for the five-layered reconstruction for channel #1 in situation 2. First column: Δ[HbX] retrieved for the upper layer; second column, the same but for the lower layer. Asterisks: actual initial values. Circles: optimum initial values.

The reconstructions shown in Sec. 4.1 were performed with the actual initial absorption coefficients. In order to test the robustness of the layered models to retrieve chromophores concentration changes when using different initial values, we spanned a space of initial $\mu_{a,U}$ and $\mu_{a,L}$ of ±50% the actual ones. The RMSEs for the particular case of the analytical five layered reconstruction are shown in Fig. 10 (top and bottom rows, Δ[HbO] and Δ[HbR]; left and right columns, upper and lower layers), where the true initial values are marked with an asterisk, while the coordinates that minimize the expression (18) are denoted with an open circle. Ideally, both marks should coincide; here, however, we notice that they seem to lie rather far apart from each other, a behavior that can be attributed to the noise in the MC simulations (specially for the long channels). In the case of the upper layer, the difference in using the true or the optimal initial values is particularly sensitive to the lower layer’s initial absorption. Then, it can be seen that simultaneously minimizing the RMSE for both species in the upper layer requires the proper knowledge of this specific initial parameter. In the case of the HbX species in the lower layer, using the real or the optimal initial parameters is more or less indistinct, since both marks are placed in the bluish region (low RMSE values); as a matter of fact, a good choice here (assuming a complete lack of knowledge) is setting $\mu_{a,U}$ and $\mu_{a,L}$ rather low. Regarding the color scales, the difference between the minimum and maximum RMSE values is around 20% for the worst case ($\Delta [\mathrm{HbO}{]}_{U}$ in Fig. 10), which implies that blindly using wrong initial coefficients of up to 50% apart from the real ones do not propagate large errors in the overall reconstruction.

#### 4.3.2 Dependence on the layers’ thicknesses

**Fig. 11:**
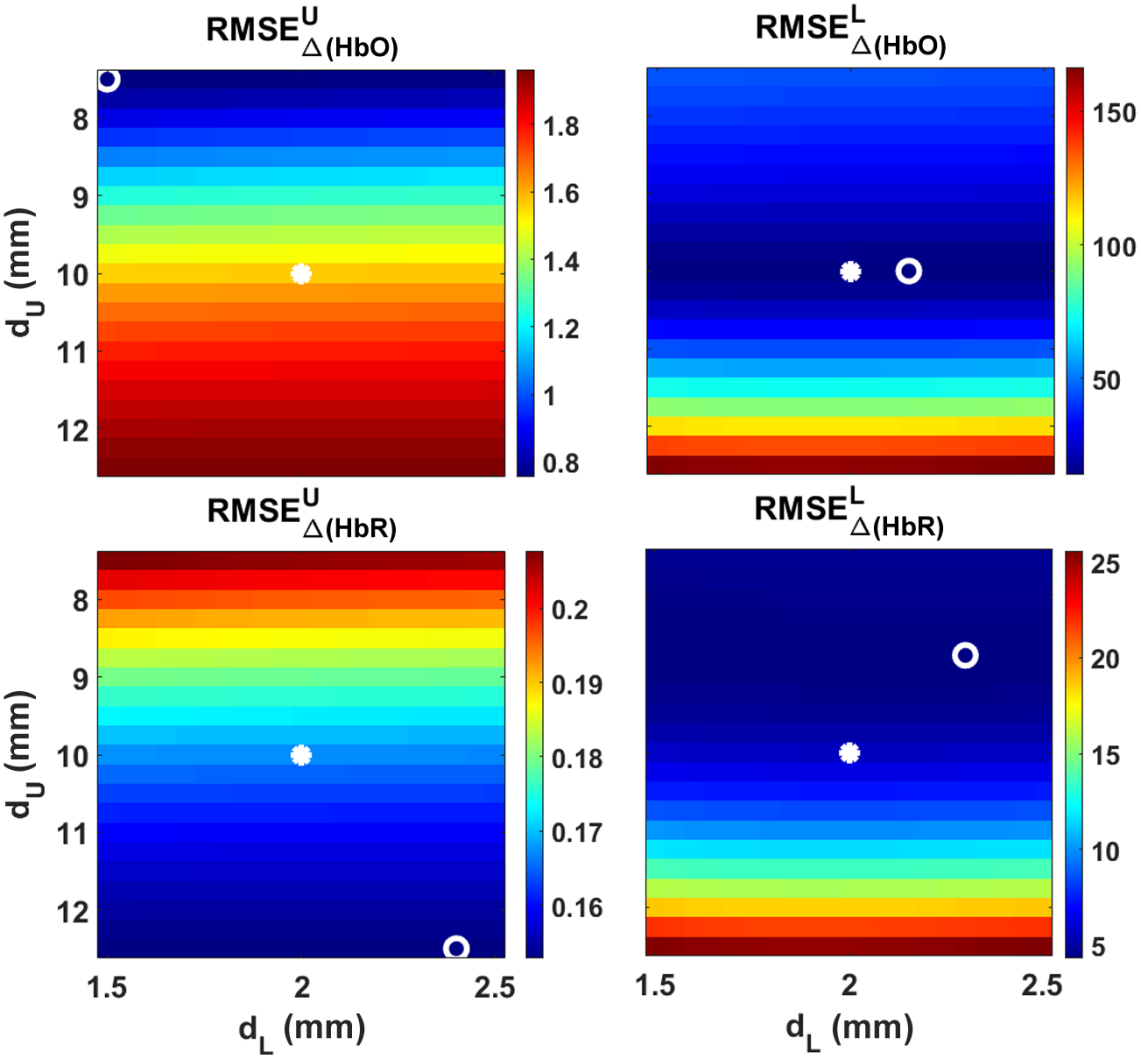
RMSE values obtained by varying the thicknesses of the upper ($d_{U}$) and lower ($d_{L}$) layers (±25% with respect to the true values) for the five-layered for channel #1 reconstruction in situation 2. First column: Δ[HbX] retrieved for the upper layer; second column, the same but for the lower layer. Asterisks: actual initial values. Circles: optimum initial values.

Figure 11 shows the RMSE for the five-layered theoretical reconstruction as computed with Eq. (18) when varying the thicknesses of the upper and the lower layer ±25% with respect to the real values (the plot order is the same and in Fig. 10). The Δ[HbX] retrievals barely depend on the lower layer’s thickness. Here, we note that for the lower layer the actual initial thicknesses are very near to the optimal ones. The difference in the RMSE index for $\Delta {\left[\mathrm{HbO}\right]}_{L}$ is just of 0.02  μM (13.96  μM versus 13.94  μM, respectively) and for $\Delta [\mathrm{HbR}{]}_{L}$ this discrepancy is of 1.31 (5.63  μM versus 4.32  μM, respectively).

As before, a good approach here would be to know and use the actual thicknesses in the model. In all cases, the effect of introducing wrong thicknesses (by ±25%) to compute the five-layered MPPLs leads to errors that are similar in magnitude to those obtained by varying the initial absorption coefficients (by ∼20%).

**Fig. 12:**
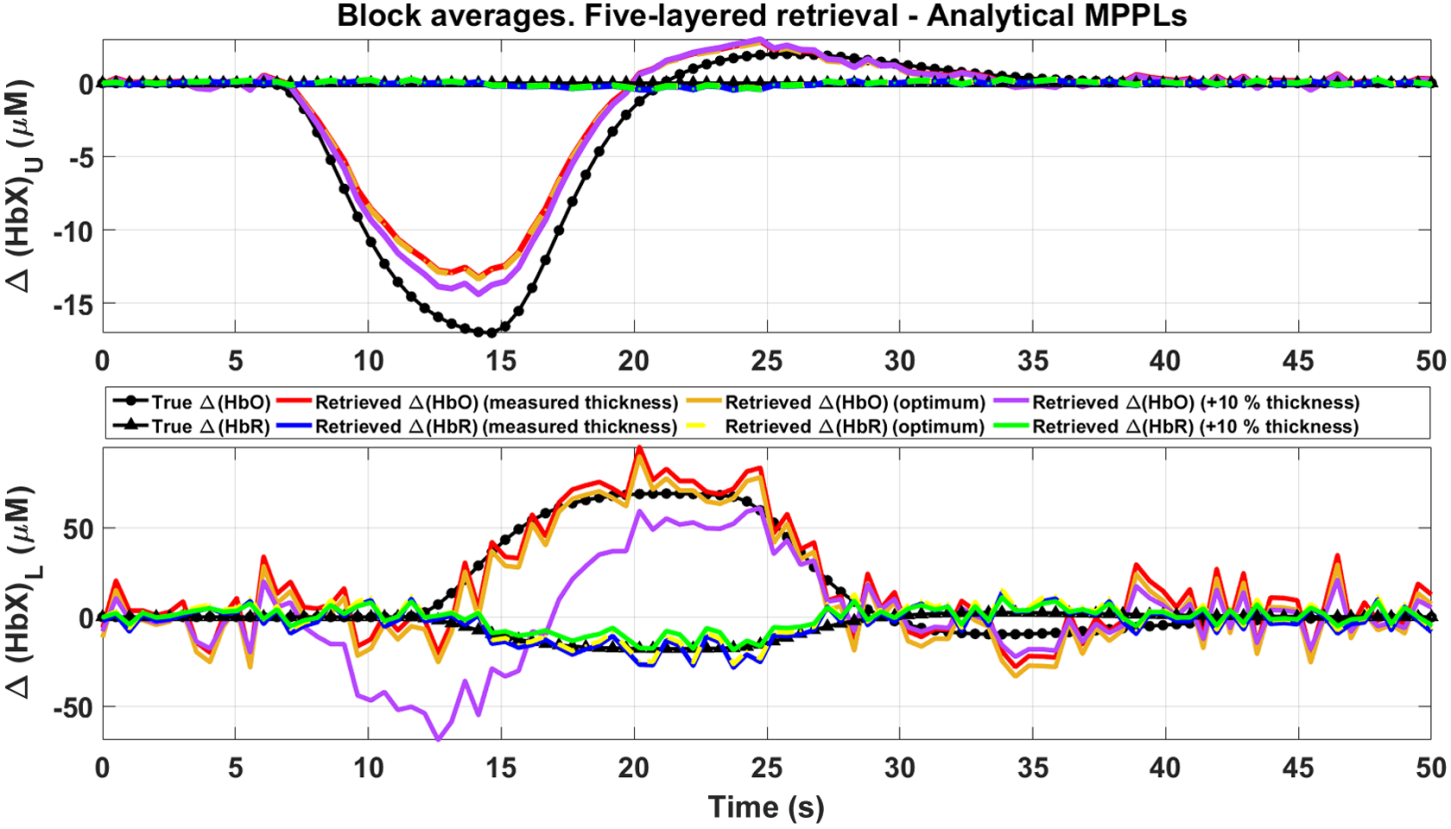
Five-layered retrieval of Δ[HbX] for channel #1 in situation 2 for different thicknesses values of the upper and lower layers (error bands removed for clarity). The red and blue lines are the retrievals obtained using the measured thicknesses. The retrievals that minimize RMSE (yellow and golden lines) as well as those obtained by varying by 10% the measured thicknesses (purple and green lines) are also included.

Figure 12 shows an example of how using different thicknesses impacts on the Δ[HbX] reconstruction in each layer (shaded error bands were removed for the sake of clarity). As usual, the black lines and symbols represent the proposed changes, while the red curves represent the retrieved changes using the actual thicknesses ($d_{\mathrm{SC}}=10\;\mathrm{mm}$ and $d_{\mathrm{GM}}=2\;\mathrm{mm}$, as stated in Table 1). As it can be seen in the top plot, the retrieval in the first layer is almost insensitive to the thicknesses chosen. This still holds true for the lower layer when comparing the real versus the optimum ($d_{\mathrm{SC}}=10\;\mathrm{mm}$ and $d_{\mathrm{GM}}=2.1\;\mathrm{mm}$) thicknesses; however, a difference of 10% in these values ($d_{\mathrm{SC}}=11\;\mathrm{mm}$ and $d_{\mathrm{GM}}=2.2\;\mathrm{mm}$) has a strong effect, notoriously visible in the Δ[HbO] signal, in particular by showing a clear undershoot at around 10 to 15 s, which is not present in the objective signal. Hence, we conclude that the thicknesses of these layers must be carefully chosen in real situations, which imply that prior information regarding the anatomy of the subject has to be taken in consideration whenever available.

### 4.4 Computational Cost

**Fig. 13:**
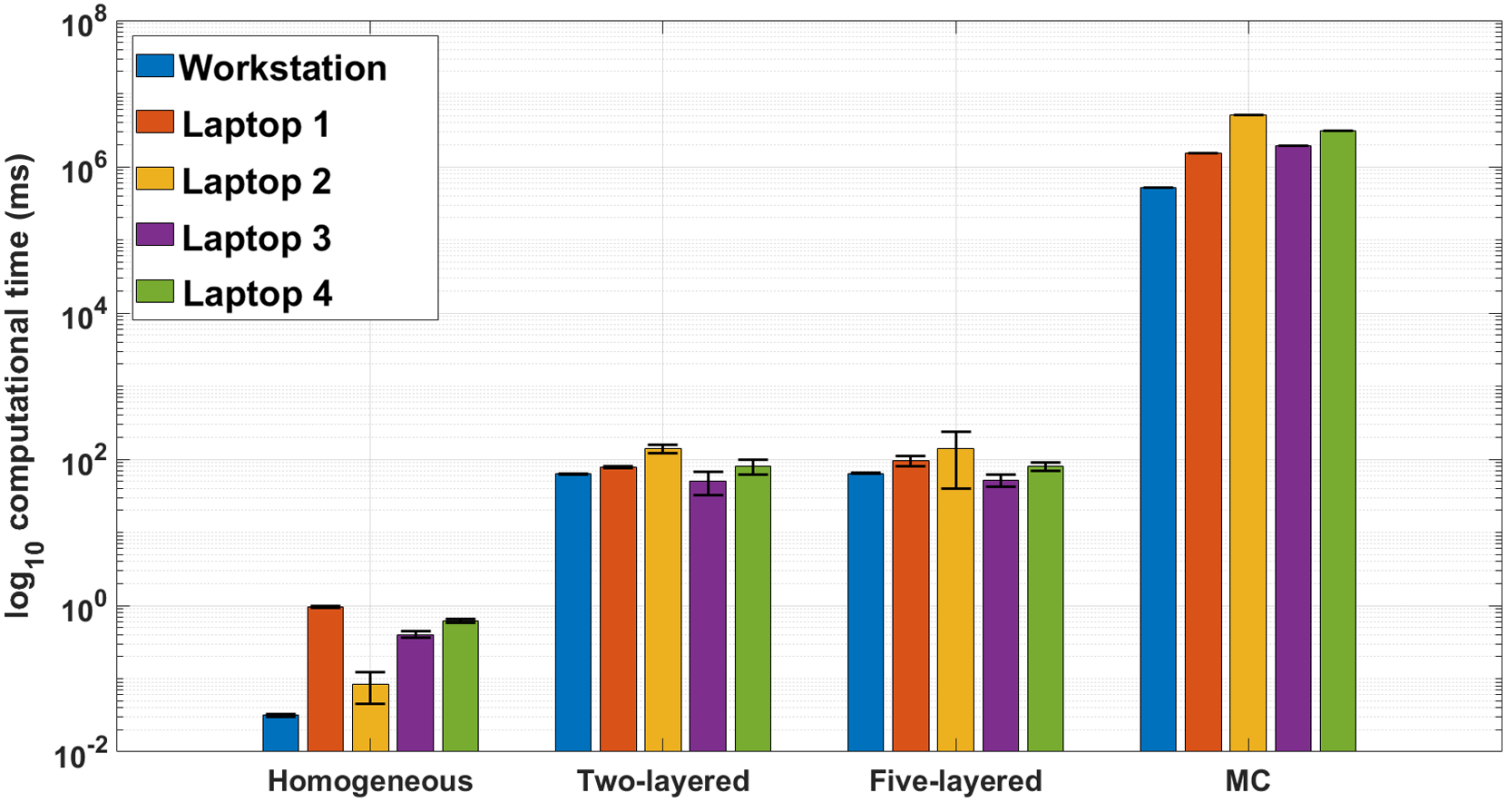
Comparison (in logarithmic scale) of the computation times taken for all models used in this work and run in five different workstations (the corresponding computing capabilities are listed in Table 4). The black bars indicate the corresponding standard deviations.

The computation times needed to perform each of the four reconstructions shown in this work are depicted in Fig. 13, for five different workstations: the one used throughout the present manuscript (simply denoted as “workstation”) used to perform all the MC simulations, whose details are given in Sec. 3; and four individual laptops with different computing capabilities (their specifications are listed in Table 4). As it can be seen, the calculations needed to obtain the MPPLs using the two- and five-layered models take, on average, 80 to 100 ms using any laptop, versus 60 ms using the workstation. The homogeneous reconstruction (corrected signal by using short channels) takes in general less than 1 ms for any of the computers. The time required to compute the MC-MPPLs is four to five orders of magnitude larger than the time needed to perform the analytical reconstructions. Although these times can be reduced by decreasing the amount of initial photon packets (at the cost of worsening the MC statistics), they still remain much larger than their analytical counterparts. Note that without the appropriate parallel computing implementation, these times are several orders of magnitude greater, a drawback that none of the analytical approaches introduced here has. Once the MPPLs have been computed, the reconstruction using either analytical MPPLs or MC’s MPPLs take the same time. Here it is worth mentioning that the codes used to compute the analytical MPPLs can still be parallelized, meaning that the corresponding computation times can be further reduced.

**Table 4:** Specifications of the workstations used to benchmark the four approaches shown in this work.

	CPU	RAM	GPU	CUDA cores
Workstation	8-core AMD Ryzen 7 5700X	64 GB	Titan XP	3840
Laptop 1	single core Intel i5-11400H	16 GB	3050 Ti	1280
Laptop 2	2-core Intel i5-72000	12 GB	Geforce 940MX	384
Laptop 3	8-core Inter i7-11370H	64 GB	Geforce RTX 3050	1024
Laptop 4	8-core Intel i7-7700HQ	16 GB	Geforce GTX 1050	640

## 5 Conclusions

In this work, we proposed a method for retrieving MC-simulated HbO and HbR concentration changes in the scalp and cortex tissues of an MRI meshed model of the human head, which is based on the use of analytical models for light propagation in turbid layered media. This approach combines the speed, flexibility, and simplicity characteristics of the most widely used homogeneous models together with the specificity and robustness of MC simulations. We tested the performance of our method using (i) a two-layered analytical model (with the two layers representing the extracerebral and the cerebral tissues) and (ii) a five-layered analytical model (accounting in this way for the scalp, skull, CSF, gray matter, and white matter). We also compared the results with the reconstructions obtained by means of the homogeneous model, as well as with MC simulations.

In all cases, the homogeneous reconstruction greatly underestimated the changes taking place in each layer when no short channels are used to correct the data, while the correction corrupted the signals to the point of destroying any sign of activation. Moreover, when the changes in both layers were shifted and had opposite signs, the uncorrected reconstruction misinterpreted the changes in the cortex tissue, associating to this layer the sign corresponding to the scalp tissue. This might be one possible cause for what can be usually found in the literature under the name of “inverse response.”^61^^–^^63^

The analytical two- and five-layered reconstructions showed similar performances when retrieving the chromophores concentration changes in the upper layer (with underestimations of the order of 20% for HbR and 30% for HbO), but the latter was much more appropriate when considering the chromophores in the gray matter, implying that the closer the number of layers in the forward model to the objective medium, the more accurate the results. It is important to notice that the layered models can correctly account not only for simultaneous information of both layers (something impossible with homogeneous models), but also for differences in signs and timing between them. Moreover, the five-layered approach is not misled by the influence of the superficial layer, especially when no changes in the cortex layer are produced, as can be concluded by comparing Figs. 8 and 9.

We also studied the robustness of the retrieving process when changing input parameters in the forward models, such as the initial absorption coefficients of the upper and lower layers, as well as their corresponding thicknesses. The accuracy of the method seems to be independent of the parameters associated with the upper layer, i.e., setting initial $\mu_{a,U}$ values ±50% apart from the real ones, as well as $d_{\mathrm{SC}}$ values ±25% apart from the actual scalp thickness, still keeps the goodness of the retrieval. On the contrary, altering the parameters associated with the lower layer may increase the error with respect to the proposed changes. In the case of the absorption coefficients, and assuming a lack of *a priori* knowledge in this regard, Fig. 10 suggests that using low initial $\mu_{a}$ values for the gray matter layer would be a good strategy; a similar approach could be used to select the value of $d_{\mathrm{GM}}$, i.e., if we are unaware of its real value, we should set it rather low (between 1.6 and 2.2 mm, according to the results shown in Fig. 11).

Although we strongly believe this contribution will encourage further improvements in fNIRS data processing and analysis strategies, a substantial amount of work needs still to be done. In the first place, the study introduced here is based on only one digital head model in a very specific brain region. Then, the results shown in this paper should be extended to other head models and brain regions. Moreover, real clinical fNIRS data acquired on humans and already validated with complementary techniques (such as MRI) could be tested with the layered models discussed in this work.

Second, we assumed optical properties for the CSF compatible with the diffusive regime, something far from reality.^38^^,^^48^^,^^49^^,^^64^ The influence of the CSF (as a thin and irregular tissue region with low light absorption and scattering surrounded by other highly diffusive layers) on NIR light propagation is still under debate. A more thorough study should include these characteristics in the forward model, being one way the derivation of the MPPLs through higher order solutions to the RTE^65^^–^^67^.

As it can be seen from the fNIRS literature,^5^^,^^13^^,^^68^ the reduced scattering coefficient is always assumed to remain constant throughout the whole measurement process, and also to take approximately the same value ($\mu_{s,j}^{\prime }∼1\;{\mathrm{mm}}^{-1}$) for all the layers. Here, we instead fed the reconstructions with the same $\mu_{s,j}^{\prime }$ values used to generate the synthetic data. Hence, a further work should include a study on how the retrieval of chromophores concentration changes is affected by using wrong values of the reduced scattering coefficients.

It must be emphasized that no data preprocessing techniques (such as smoothing or noise filtering) were implemented in any of the situations and reconstructions discussed in this work, but the algorithms were always fed with purely raw data instead. Appropriate preprocessing strategies could be applied in further studies to improve the reconstruction, but this can be also complemented with the artificial addition of physiological signals (such as those coming from heart and breathing rates, together with Mayer waves) or artifacts due to motion.^69^

The appealing simplicity of planar interfaces between layers led us to ignore the curved geometry of the human head in the forward model. However, the reconstruction performed with MC simulations showed that a proper interface description plays a relevant role. Although spherical geometries are still far from the actual irregular limits between tissue layers, they could imply a fair improvement in the way we describe light propagation in tissue.^70^

Finally, the model described in this paper is based on a CW approach (which is associated to non-expensive fNIRS devices and technologies) and can be easily implemented in any of the current several data processing/analysis tools available.^50^^,^^58^^,^^71^^,^^72^ However, TD techniques are gaining even more interest in the fNIRS community due to the possibility of measuring much more useful information.^26^^,^^28^^,^^73^^,^^74^ This implies that, eventually, analytical TD-MPPLs might be developed to assist TD-based devices in real time and to understand how the time-of-flight of photons can shed light on brain hemodynamics.

## Data Availability

Data underlying the results presented in this paper are not publicly available at this time but may be obtained from the authors upon reasonable request.
